# ARADEEPOPSIS, an Automated Workflow for Top-View Plant Phenomics using Semantic Segmentation of Leaf States

**DOI:** 10.1105/tpc.20.00318

**Published:** 2020-10-09

**Authors:** Patrick Hüther, Niklas Schandry, Katharina Jandrasits, Ilja Bezrukov, Claude Becker

**Affiliations:** a Gregor Mendel Institute of Molecular Plant Biology (GMI), Austrian Academy of Sciences, Vienna BioCenter (VBC), 1030 Vienna, Austria; b Genetics, Faculty of Biology, Ludwig-Maximilians-University München, 82152 Martinsried, Germany; c Department of Molecular Biology, Max Planck Institute of Developmental Biology, 72076 Tübingen, Germany

## Abstract

Linking plant phenotype to genotype is a common goal to both plant breeders and geneticists. However, collecting phenotypic data for large numbers of plants remain a bottleneck. Plant phenotyping is mostly image based and therefore requires rapid and robust extraction of phenotypic measurements from image data. However, because segmentation tools usually rely on color information, they are sensitive to background or plant color deviations. We have developed a versatile, fully open-source pipeline to extract phenotypic measurements from plant images in an unsupervised manner. ARADEEPOPSIS (https://github.com/Gregor-Mendel-Institute/aradeepopsis) uses semantic segmentation of top-view images to classify leaf tissue into three categories: healthy, anthocyanin rich, and senescent. This makes it particularly powerful at quantitative phenotyping of different developmental stages, mutants with aberrant leaf color and/or phenotype, and plants growing in stressful conditions. On a panel of 210 natural Arabidopsis (*Arabidopsis thaliana*) accessions, we were able to not only accurately segment images of phenotypically diverse genotypes but also to identify known loci related to anthocyanin production and early necrosis in genome-wide association analyses. Our pipeline accurately processed images of diverse origin, quality, and background composition, and of a distantly related Brassicaceae. ARADEEPOPSIS is deployable on most operating systems and high-performance computing environments and can be used independently of bioinformatics expertise and resources.

## Abstract (precis)

ARADEEPOPSIS is a robust, open-source, and easy-to-use pipeline to extract plant phenotypic traits and to classify leaf states from large-scale image data.

## Introduction

### The Phenotyping Bottleneck in Plant -Omics Studies

Over the last decades, molecular techniques have steadily increased in throughput while decreasing in cost. A prime example is nucleic acid sequencing, which has followed a trend analogous to Moore’s law in computer science. However, phenotyping methods aimed at determining the physical shape of an organism and measuring morphological parameters have not kept up with this pace, which has caused a “phenotyping bottleneck” (Furbank and Tester, 2011) in the design and execution of scientific studies. This phenotyping bottleneck constitutes a major challenge in plant biology as well, due to two major issues: image acquisition and data analysis. Data acquisition in most plant phenotyping scenarios involves acquiring standardized images of plants growing in a controlled environment. Plant phenotyping requires space and a dedicated infrastructure that can accommodate the developmental and growth transitions that occur over the life of a plant. Moreover, plant development needs to be phenotyped over relatively long periods of time. For example, in the case of the model plant Arabidopsis (*Arabidopsis thaliana*), a relatively small and fast-growing species, a phenotyping experiment typically runs for several weeks or months, depending on the phenotype of interest and growth conditions. In their vegetative phase, that is, before they produce shoots, flowers, and seeds, Arabidopsis plants grow as relatively small, flat rosettes and can therefore be considered two-dimensional. Because the entire plant is visible from the top during this phase, top-view phenotyping is a straightforward method to determine size, growth rate, and leaf development. While this type of high-throughput image acquisition has become almost trivial, robustly and faithfully extracting meaningful data from these images has not.

**Figure:**
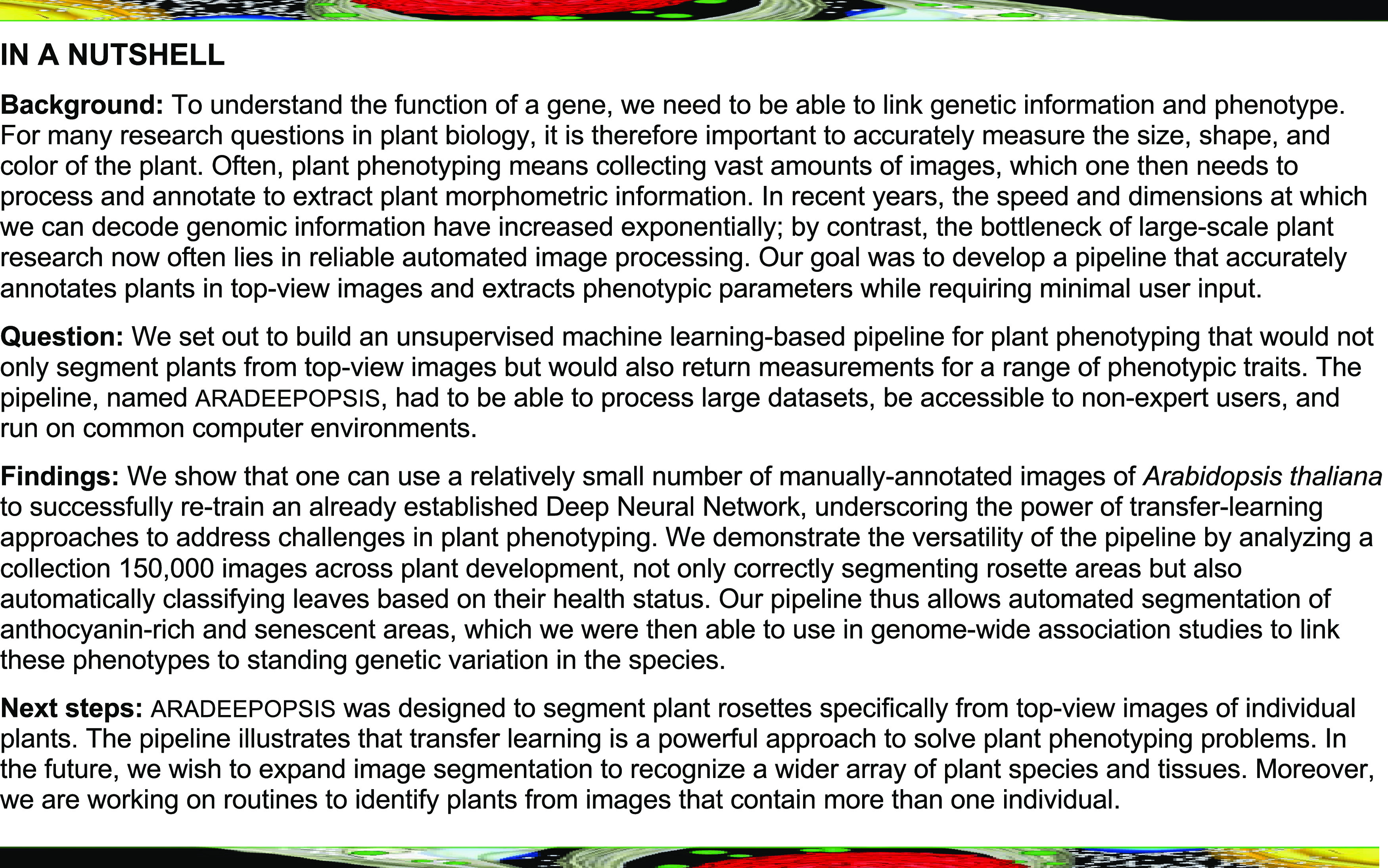


Indeed, overcoming the data acquisition challenge, by means of a dedicated plant phenotyping facility for example, does not mitigate the second cause of the phenotyping bottleneck: data analysis. In the context of image-based phenotyping data, analysis includes automated image processing for object detection, object segmentation, and extraction of quantitative phenotypic traits.

### Challenges in Image Analysis

The first key step from images to quantitative phenotypic data is also the most difficult: defining the area in the image that depicts the object of interest, in this case a plant. On a small set of images, this segmentation can be done manually by delineating the plant object using software such as *ImageJ* (Schindelin et al., 2012; Schneider et al., 2012). On data sets consisting of hundreds or thousands of images, which are typical for experiments from phenotyping platforms, this segmentation task must be automated, both to speed up the process and to neutralize user bias. Commonly used software for segmenting plant objects from digital images rely on color information. In simple terms, such color information is stored in tables, with information on each pixel stored in a corresponding cell. While grayscale images are stored in a single table, where each cell contains the grayscale value of the corresponding pixel, color image information is stored in several such tables. For images using the very common additive Red-Green-Blue (RGB) color model, three separate tables store information for the red (R), green (G), and blue (B) color channel intensities for each pixel. A very simple approach to differentiate plants from background is to assume that, since plants are green, one can assign all pixels that pass a certain threshold in the green channel to the object ‘plant’ and ignore all other pixels. This approach is called binary thresholding and works well if, and only if, the assumption is correct that all plants in the experiment are green and that the remaining area of the image is not. In reality, this assumption is often violated: plants can produce high quantities of anthocyanins under certain circumstances, which give plants a red or purple hue. Plants may also develop chlorosis or become senescent, in which case they turn yellow or brown and, ultimately, white. Moreover, as they grow larger over the course of an experiment, the leaves of neighboring plants can protrude into the image area of the monitored plant, thus creating additional green areas that should not be taken into account. Even the background color can fluctuate, either because of constraints in the experimental setup, or because other organisms, such as algae, start growing on the soil surface. In summary, color-based segmentation is sensitive to image parameters and developmental stage, which are difficult to control in a biological experiment, and therefore often needs to be verified by laborious and time-consuming visual inspection of the individual images to identify and correct erroneous segmentation and artifacts. We therefore sought to develop an alternative, unsupervised, high-throughput approach that is robust against variation in color parameters and image characteristics.

### Machine Learning Methods in Plant Phenotyping

Alternative approaches to object segmentation that solve the aforementioned problems of color-based segmentation are available: they are based on machine learning methods and include Gaussian mixture models (Rother et al., 2004) or Naïve Bayes classifiers (Gehan et al., 2017). While these approaches are highly flexible, their correct implementation on new data sets requires substantial programming knowledge and deeper understanding of the underlying statistical principles.

More recently, successful alternative approaches to segment particular classes of objects from images have come from the deep learning field. Convolutional neural networks have proven invaluable for image classification and segmentation tasks (Lecun et al., 1998; Krizhevsky et al., 2012; He et al., 2016; Chollet, 2017). They perform well on data that has a local structure, which is inherently the case in images where values of neighboring pixels tend to be highly correlated and contain recurring structures such as corners and edges. These models are supervised, meaning that they are provided with a set of manually annotated images containing the “ground truth” for each pixel, from which the model will attempt to derive rules for the classification of pixels based on the training data set. This training process is iterative and usually performed over many thousands of iterations, during which the model attempts to map input images to their corresponding ground truth annotations. In brief, during training, a loss function is calculated to estimate the error between input and output, and the model subsequently tries to minimize this error via back-propagation (Rumelhart et al., 1986). Weight matrices throughout the network layers are updated along a gradient, following the chain rule to calculate partial derivatives of the loss function with regard to the layer weights. Due to the nonconvex nature of the loss function, error minimization typically reaches only local minima, requiring careful selection of model parameters and a large and diverse set of training data to avoid overfitting. The latter is usually scarce because ground truth data for supervised learning have to be generated by meticulous manual annotation. Depending on the nature of the desired feature to be extracted, such a task is typically labor intensive and time consuming due to the large number of data points required to train a well-generalizing deep learning model de novo, and hence exceeds the capacity of many experimental research labs.

Such models have been successfully applied to plant phenotyping for both aerial and root tissue (Pound et al., 2017; Ubbens et al., 2018; Yasrab et al., 2019; Wang et al., 2020). The Deep Plant Phenomics (DPP) platform provides a framework to either build models suitable for phenotyping tasks or use pretrained models (Ubbens and Stavness, 2017). Because these models are trained from scratch, their ability to generalize to unseen data is limited by the amount of annotations contained in the training set, even if data augmentation such as random cropping, contrast, or brightness adjustments are performed. Despite the lowered bar of entry provided by DPP, training of specialized models still requires programming knowledge, which may prevent nonexpert users from applying these tools to their data.

Here, we use an alternative approach toward the application of convolutional neural networks in plant phenomics. Our aim was to lower the amount of required training data, while at the same time reaching high accuracy across images sourced from different imaging platforms. To this end, we make use of a well-established large network architecture that had already been trained on the ImageNet (Deng et al., 2009) data set, consisting of millions of annotated images. Our approach, named ARADEEPOPSIS (ARAbidopsis DEEP-learning-based OPtimal Semantic Image Segmentation), is an image analysis pipeline for unsupervised plant segmentation and phenotypic trait extraction from large image data sets. Rather than developing a new model for plant segmentation, we used transfer learning by centering our pipeline around the well-established deep-learning model DeepLabV3+, which we re-trained for segmentation of Arabidopsis rosettes in top view. Besides accurately identifying image areas containing green plants (rosettes), our pipeline also faithfully phenotypes very late developmental stages, in which plants often undergo color changes. ARADEEPOPSIS can discriminate three different health states of leaves (green/healthy, high in anthocyanins, and senescent) during image segmentation, during which it is able to accurately segment and quantify plants showing altered phenotypes arising from developmental transitions or physiological responses to environmental stresses. We show how ARADEEPOPSIS can be applied to reliably segment Arabidopsis rosettes independent of shape, age, health state, and background, which is not possible with color-based segmentation algorithms. By noninvasively extracting color index data from the segmented leaf area, our tool delivered highly resolved quantitative data that we successfully applied to a genome-wide association (GWA) analysis of anthocyanin content. ARADEEPOPSIS succeeds in delivering quantitative data on the notoriously difficult phenotype of plant senescence from the analysis of plant images, from which we identified genetic variants that drive premature senescence. Because Arabidopsis is a widely used model species in molecular plant biology and genetics, we centered our pipeline on this species. Additionally, we show that it can accurately segment other species.

ARADEEPOPSIS requires minimal user input compared to other tools that offer semi-automated analysis; it is open source and available for free. ARADEEPOPSIS provides a convenient all-in-one solution for machine learning-based image analysis and trait-extraction and significantly lowers the bar to nonbioinformaticians who may wish to apply machine learning for plant phenotyping. Because the pipeline is written in *Nextflow* and supports containerization, researchers with little computational background can install and run it on their personal computer, cloud infrastructure, or in a high-performance computing environment. ARADEEPOPSIS is available at https://github.com/Gregor-Mendel-Institute/aradeepopsis.

## Results

### Color-Guided Segmentation Can be Misguided by Image and Object Parameters

**Figure 1.**
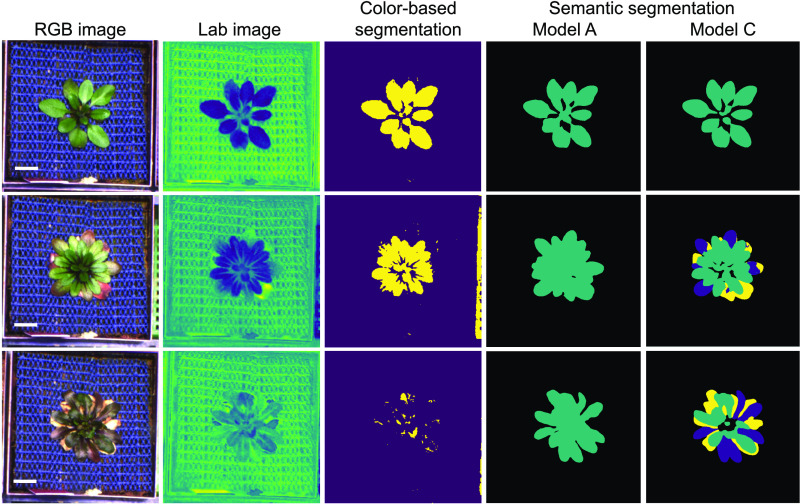
Performance of Color-Based Versus Semantic Segmentation. The left-most column shows the original RGB images of a representative Arabidopsis plant from our phenotyping experiment at three different developmental stages. The second column shows the same images transformed into the` Lab color space, which enhances contrast between green and other colors. Binary thresholding (third column), based on Lab input and used for color-based segmentation, results in difficulties to correctly segment older or anthocyanin-containing plants. Semantic segmentation by our models A and C is insensitive to color and contrast of the plant (two right-most columns). For an explanation of the model C color code, see Figure 2. Scale bar = 1 cm.

We wished to explore the performance and accuracy of classical color-based segmentation and of a self-learning algorithm for segmenting Arabidopsis rosettes from a large, automated phenotyping experiment. We monitored the growth of 210 Arabidopsis accessions from the 1001 genomes panel (The 1001 Genomes Consortium, 2016) in natural soil, under climate-controlled conditions, with six replicates per genotype, from seed to flowering stage. Using an automated phenotyping system, we recorded top-view images twice per day, cropped them to frames containing single pots, and subjected them to rosette area measurements. We first used *LemnaGrid* (Lemnatec), which, similar to other integrated pipelines that perform (semi)-supervised segmentation, relies on color channel information to segment plants in top-view images and is commonly used for Arabidopsis phenotyping (Arvidsson et al., 2011; Minervini et al., 2017). The implementation of this pipeline resulted in the accurate segmentation of young and healthy plants that were mainly composed of green tissue (Figure 1). However, because the plants in our data set were grown over a long period in natural soil, many accumulated high levels of anthocyanins in their leaves, either because of genetic determinants or as a general stress response to the natural soil and its abiotic or biotic composition; others showed an onset of senescence at later stages of the experiment. For such images, segmentation often failed completely or resulted in only partial or inaccurate segmentation of the plant (Figure 1), showing that color-based segmentation is sensitive to deviations from the expected color composition of the object to be detected.

### Training of a Proof-of-Principle Model

We hypothesized that self-learning algorithms would achieve higher accuracy while at the same time be more robust against shifts in color patterns. In a process referred to as transfer learning, trained model architectures that do well at pixel classification can be re-trained on new data that contain new classes while retaining already trained weights in layers that extract low-level features such as shapes and edges. In other words, a model may be re-trained on plant rosettes even when the initial data set the model was trained on does not contain plant rosettes. Model design is a complex task, and experts in the field of machine learning have invested considerable effort into establishing sophisticated model architectures that can be built upon. Moreover, because re-training a model mainly consists of updating the last layer of the model, transfer learning on a model that already performs well at image segmentation is much faster and requires less training data than designing and training a new model from scratch, since the established model is already able to extract features from image data and is merely learning a new classification output (Razavian et al., 2014).

To test whether transfer learning of the well-established segmentation model DeepLabV3+ would result in a model that could faithfully extract Arabidopsis rosettes from top-view images, independently of developmental stage or phenotype, we generated a 'ground-truth' training set. We generated a small data set of 300 images, in which we manually annotated the rosette area, deliberately excluding senescent leaves. From these 300 annotations, we randomly selected 80% (240 images) for model training and kept the remaining 20% for evaluating the accuracy of the new model.

After training, this model (referred to hereafter as model A) performed well on plants at various developmental stages and with different leaf coloring (Figure 1), which encouraged us to generate a much larger ground-truth data set for more fine-grained models (https://doi.org/10.5281/zenodo.3946393).

### Advanced Models for Differentiated Plant Area Classification of Individual Leaves

From the example images shown in Figure 1, it is apparent that plant health changes not only in the context of the whole rosette but also between individual leaves or parts of leaves, with some accumulating high anthocyanin levels and others entering senescence. We therefore asked if we could train a model that would semantically extract such features from leaves and assign them to different classes, depending on their phenotypic appearance. Not only would such a model allow finer resolution when assessing the overall health of the plant, it would also enable segmenting differently colored areas.

**Figure 2.**
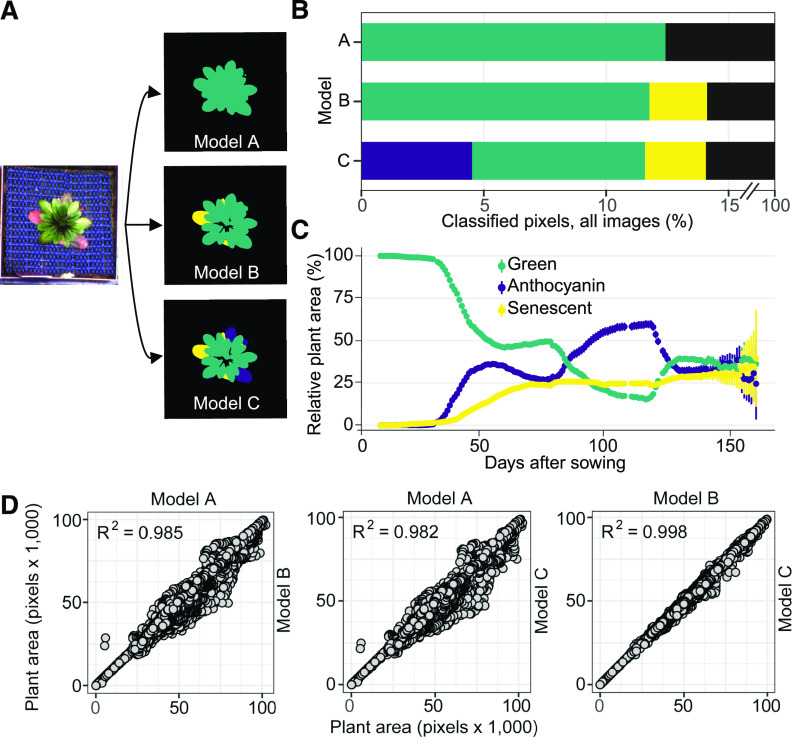
The Three Default Models Available Through ARADEEPOPSIS. **(A)** Example of segmentation results from the one-class model A, the two-class model B, and the three-class model C on a single Arabidopsis individual. **(B)** Comparison of the percentage of classified pixels across all 148,792 images between the three available models (colors as in C; black = background). **(C)** Relative number of pixels classified as green, anthocyanin-rich, or senescent by model C over time. The mean percentage of pixels assigned to each class is shown per day. Error bars indicate 95% confidence intervals. **(D)** Scatterplots showing pairwise correlations between measurements of plant area (includes all leaf states except senescence) between models A, B, and C.

Reasoning that it should be possible to segment senescent leaves separately, we generated a second, larger, and more finely annotated training data set, consisting of 1375 manually annotated top-view images of Arabidopsis plants from the phenotyping experiment described above, from which we again withheld 20% for later validation. The images used for annotation were selected semi-randomly such that they covered various developmental stages and included healthy-looking as well as stressed plants from different genotypes that exhibited altered color profiles due to anthocyanin accumulation or senescence, and that were diverse in leaf-morphology. Instead of manually annotating the entire rosette, as we had done for the initial training set, we annotated all leaves of each rosette individually and manually assigned each leaf to one of three different classes, depending on its appearance: green; anthocyanin-rich; senescent or dead. From these annotations, we generated image masks for two additional models complementing the initial one-class model A. For the two-class model B, we classified senescent (class_senesc) versus nonsenescent leaves (class_norm), whereas the three-class model C was trained to differentiate between senescent (class_senesc), anthocyanin-rich (class_antho), and green (class_norm) areas (Figure 2A). We then used these three annotated sets of masks for transfer learning of a publicly available *xception65* (Chollet, 2017) based DeepLabV3+ checkpoint that had been pretrained on the ImageNet data set (see Methods and https://doi.org/10.5281/zenodo.3946618; Chen et al., 2017; Chen et al., 2018).

After training of all three models had completed, we assessed their performance according to the mean intersection over union (mIoU) for all pixel-level classifications, which is defined as the mean fraction of true positives divided by the sum of true positives, false positives, and false negatives over all annotated classes. When applied to the validation data set, models A, B, and C reached an mIoU of 96.8%, 84.0%, and 83.1%, respectively.

Next, we compared how each model performed at segmenting the 148,792 rosette images comprising our data set. First, we asked how much the different classes contributed to the area segmented as 'plant'. Averaged across all images, model A, which ignores senescent leaves, classified 12.5% of image area as plant tissue (Figure 2B). Models B and C both classified ∼14% of image area as plant tissue (including senescent areas ignored by model A; Figure 2B). The fraction of class_senesc segmentation was almost identical in models B and C. To further test whether our most complex model, model C, performed well, we analyzed the ratio of the three classes over the course of the experiment. As expected, we saw a sharp increase in anthocyanin-rich area beginning 30 d after sowing, followed by an increase in senescent segmentations 10 d later (Figure 2C). Relative classification of green tissue decreased accordingly. This observation demonstrated that model C successfully captured an increase in senescence as plants grew older, accurately reflecting plant development.

Next, we determined whether the three models behaved differently in segmenting the combined rosette area. Using the total pixel area classified as leaves from each image, we found that models B (two-class) and C (three-class) displayed the strongest correlation (R^2^ = 0.998; Figure 2D). Model A, our one-class model trained on annotations of whole rosettes rather than individual leaves, correlated slightly less well with models B and C (R^2^ = 0.985 and 0.982, respectively) and had a tendency to segment larger areas than either of the other two models (Figure 2D). Visual inspection of overlays of the segmentations produced by model A with the original image revealed that segmentations produced by model A frequently included areas between leaves that were ignored by models B and C, explaining the larger rosette sizes measured by model A (see exemplary segmentations in Figure 2A). We believe that these differences are related to the different annotation strategies (whole rosettes in model A vs. more refined per-leaf annotations for the larger data sets in models B and C). When generating the ground-truth training set for models B and C, we noticed how nontrivial it was to make consistent decisions as to when a leaf should be annotated as senescent or rich in anthocyanins, as the transitions are gradual, hence classification can become subjective. This lack of total precision during annotation of the training set might also explain the decrease in mIoU scores that we observed with the two- and three-class models. We attempted to mitigate user bias by having several individuals contributing to generating the annotations for a subset of images, thus ensuring that the model would faithfully learn the features of interest.

### Extraction of Morphometric and Color Index Traits from Segmented Images

**Figure 3.**
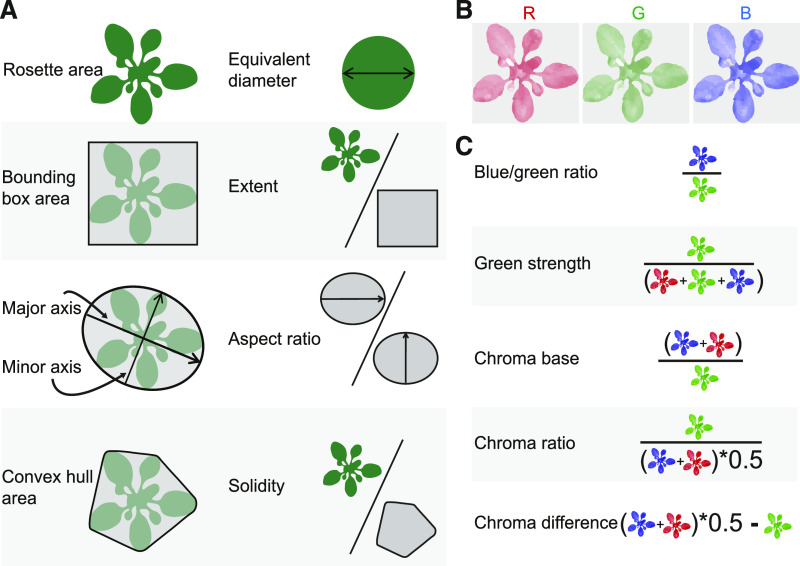
Morphometric and Color Index Measurements Extracted from Segmentation Masks. **(A)** Morphometric traits extracted using the Python library *scikit-image* (van der Walt et al., 2014). **(B)** Separation of the segmented plant image into red **(R)**, green **(G)**, and blue **(B)** color channels. **(C)** Color channel indices calculated as described in Del Valle et al. (2018). The differently colored plants represent mean values of the different color channels; see **(B)**.

Next, we used the segmentation masks obtained from the models created by transfer learning to extract morphometric and color index traits associated with each plant (Figure 3), using the Python library *scikit-image* (van der Walt et al., 2014; Del Valle et al., 2018). Besides the total number of pixels annotated as 'plant', the pipeline returns the equivalent diameter, that is, the diameter of a circle with the same number of pixels as the plant area. The major and minor axes of an object-surrounding ellipse are a measure of the aspect ratio of the plant and inform on whether the rosette is rather round or elongated along one axis (Figure 3A). The area of a convex hull surrounding the object is a measure of plant size; the solidity of the plant object is calculated by dividing the area of the plant by the area of the convex hull. Finally, dividing the plant area by the area of its bounding box, that is, a minimal box enclosing the object, is representative of the overall extent of the plant (Figure 3A). Depending on the type of downstream analyses, these indirect object parameters can be used as proxies for overall or specific growth.

The segmentation masks are also used to extract color index information from the plant area (Figure 3B). These traits are based on the intensity of each RGB color channel per pixel. Simple color index traits are, for example, the average intensity of the green channel over all pixels classified as 'plant.' We have implemented the color index traits described by Del Valle et al. (2018) in our pipeline (Figure 3C). These traits are calculated for each class individually and for a compound class termed “plant_region,” which contains both class_norm and class_antho (only for model C). Details on the color index traits are shown in Figure 3C and provided in Methods.

### The ARADEEPOPSIS Pipeline

**Figure 4.**
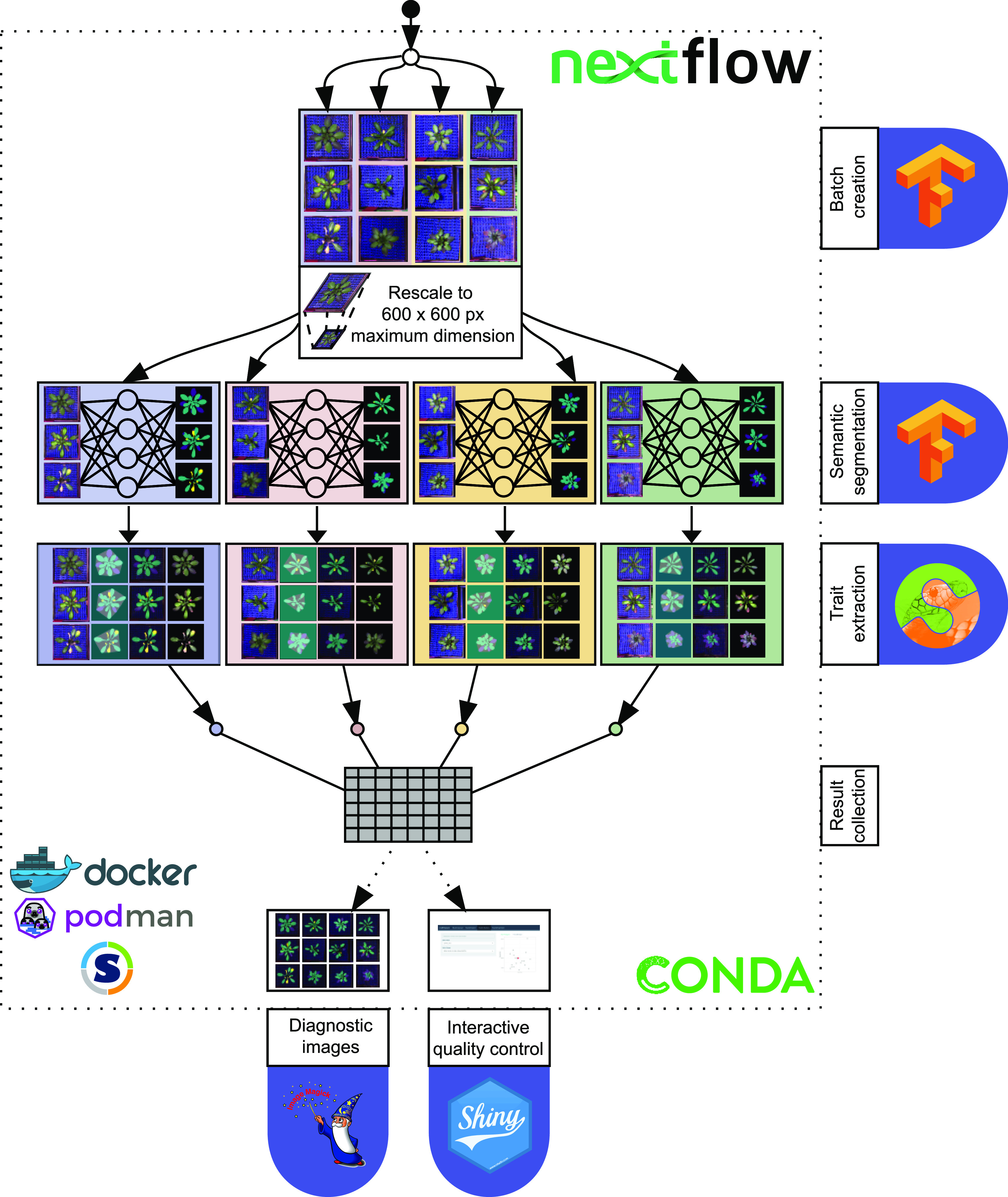
The ARADEEPOPSIS Pipeline. A folder containing image files is passed to the pipeline. The total number of images is split into batches of a fixed size (indicated by different background colors). Batches are processed in parallel: first, the segmentation is performed on each batch, then traits are extracted from the segmented images. The output from all batches is collected in one final table, the results table. In addition, the pipeline produces diagnostic images that show the segmentation results overlaid on the original image, color-coded segmentation masks, and background-subtracted plant rosettes. These diagnostic images can be explored in a *Shiny* (RStudio, 2014) app, which is launched at the end of an ARADEEPOPSIS run, such that users can visually inspect the segmentations and verify their accuracy.

The ARADEEPOPSIS (ARAbidopsis DEEP-learning-based OPtimal Semantic Image Segmentation) pipeline integrates the unsupervised image segmentation with the trait extraction outlined above. The segmentation results are easily browsable with a custom *Shiny* application, allowing for straightforward quality assessment of the obtained results. ARADEEPOPSIS is written in *Nextflow* (Di Tommaso et al., 2017), a high-level data-science language used to implement bioinformatics workflows and geared toward increasing portability of these workflows across different hardware and scheduling systems. The pipeline is fully open-source and licensed under GPLv3; the technical details are laid out in the “Methods” sections. Briefly, the pipeline takes a folder of images as an input, splits the total image set into batches of equal size, and performs semantic segmentation on the images using a model of choice (see section Advanced Models for Differentiated Plant Area Classification of Individual Leaves). The semantic segmentation output is then used to extract morphometric and color index traits from each individual image (see section Extraction of Morphometric and Color Index Traits from Segmented Images). Quality control and exploratory analyses can be performed after the pipeline has finished by launching a bundled *Shiny* (RStudio, 2014) application. In addition to offering some straightforward visualization of the results, *Shiny* also provides an interface to merge metadata and ARADEEPOPSIS output for downstream analysis (Figure 4; Di Tommaso et al., 2017). ARADEEPOPSIS is built modularly, and trait extraction works independently of the model: that is, although our pipeline comes with the three described models for plant segmentation (models A, B, and C; Figure 2), it offers flexibility for experienced users, via the option to feed in custom *TensorFlow* models (e.g., created using DPP). Alternatively, users can choose to use only the trait extraction and visualization steps of the pipeline by providing their own segmentation masks along with the original images.

ARADEEPOPSIS is designed to segment plants from images that contain only a single plant, and we do not recommend usage on images containing multiple plants. In case the phenotyping system records whole or partial trays of plant pots, as is common for many automated phenotyping platforms, users will need to preprocess these images and divide them into sub-images, each containing a single individual. Unless explicitly stated otherwise, we use ARADEEPOPSIS to refer to the pipeline using model C (Figure 2) from here on.

### Validation and Generalization to Public Phenotyping Data Sets and Comparison to Other Models

To validate the accuracy of our pipeline, we re-analyzed publicly available images and compared the output of ARADEEPOPSIS to the published analyses. This validation step served two purposes: first, we wished to confirm that the rosette features extracted by ARADEEPOPSIS segmentation were accurately reproducing published data. Second, we wanted to test whether our pipeline would remain robust when using data generated on different phenotyping platforms, with different image recording systems, pot sizes and shapes, and background composition. We used images from three published studies (Baute et al., 2017; Capovilla et al., 2018; Barragan et al., 2019), generated on either the RAPA platform (Vasseur et al., 2018) at the Max Planck Institute for Developmental Biology in Tübingen, Germany (Capovilla et al., 2018; Barragan et al., 2019), or the Weighing, Imaging and Watering Machine platform (https://www.psb.ugent.be/phenotyping-platforms) at the VIB in Ghent, Belgium (Baute et al., 2017; Coppens et al., 2017), and re-analyzed them using ARADEEPOPSIS.

**Figure 5.**
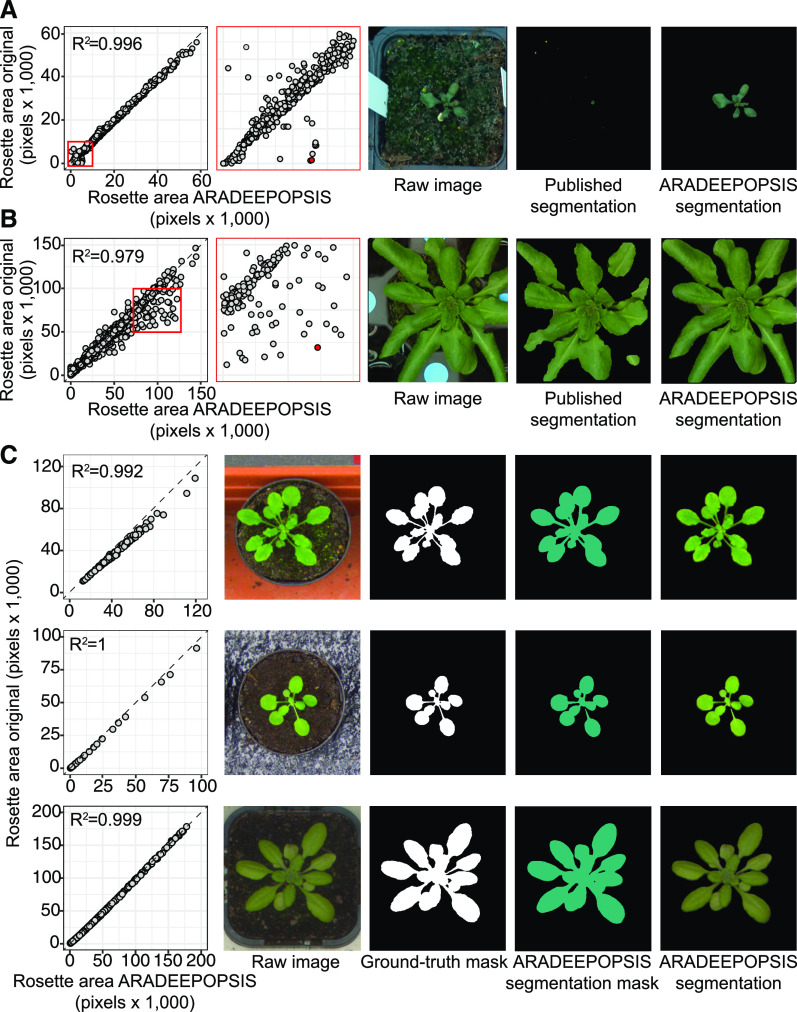
Validation of ARADEEPOPSIS Output Against Published Data. **(A)** Validation against data from Capovilla et al. (2018). The left-most panel shows the correlation between values produced by ARADEEPOPSIS against published data; the boxed area is magnified in the second panel to highlight disagreements. The third panel shows the original image corresponding to the red data point in panel two. Panel four shows the original segmentation, panel five the segmentation by ARADEEPOPSIS. **(B)** Validation against Barragan et al. (2019) with the same order as in **(A)**. **(C)** Validation against the A1 (top row), A2 (middle), and A4 (bottom) Arabidopsis image series (A1, A2, and A4) from the IPPN data set (Minervini et al., 2016). The ground-truth mask corresponds to the manually annotated foreground mask provided by IPPN.

Correlation analysis of the published rosette area derived from the RAPA system and our own measurements resulted in R^2^ values of 0.996 (Capovilla et al., 2018) and 0.979 (Barragan et al., 2019), respectively (Figures 5A and 5B). Despite this overall very strong correlation, we noticed individual images in which our segmentation disagreed with the published record. When inspecting the respective images and segmentation masks from both analyses, we discovered that the respective rosette segmentations in the original analysis were inaccurate or incomplete, and that ARADEEPOPSIS segmentation was in strong agreement with the actual rosette in the image, even when plants had strong aberrant phenotypes or grew on low-contrast background (Figures 5A and 5B). We also used the pretrained *vegetationSegmentation* model from DPP to segment these two data sets and noticed a markedly lower to between DPP-derived and published measurements (Supplemental Figure 1A) when compared with ARADEEPOPSIS.

Re-analysis of the Weighing, Imaging and Watering Machine data set (Baute et al., 2017) also showed high correlation between the published measurements and our analysis (Supplemental Figure 1B). Unfortunately, original segmentations were not available for these images, but when inspecting some of the outliers, we again noticed highly accurate segmentation by ARADEEPOPSIS relative to the actual image (Supplemental Figure 1A).

To further validate the ARADEEPOPSIS pipeline, we assessed its performance on the International Plant Phenotyping Network (IPPN) data set (https://www.plant-phenotyping.org/data sets-download; Minervini et al., 2016), which contains diverse images of plants with a range of backgrounds and varying image qualities. Importantly, this data set was not part of the training data set used during transfer learning. We used the ground-truth annotation masks of the IPPN data set with ARADEEPOPSIS to extract the respective ground-truth traits. We found that the models provided by ARADEEPOPSIS generalized well to this data set, although we noticed that some of the models (B and C) occasionally segmented background into classes corresponding to anthocyanin-rich or senescent, or misclassified algae as leaf tissue (Supplemental Figure 2B). Such artifacts can be reduced by running ARADEEPOPSIS with multi-scale inference, which improves segmentation accuracy by scaling the input image by different scale factors before model prediction and taking the average over these multiple scales for classification of individual pixels. With multi-scale inference, the segmentations of green plant tissue (ARADEEPOPSIS model C) agreed with the ground-truth annotations available for the IPPN data set (Figure 5C) and correlated better than those obtained when running our pipeline with the pretrained DPP vegetation segmentation model (Supplemental Figure 3). Because multi-scale inference results in higher computational demand, single-scale inference is the default setting; users will need to choose based on the availability of computational resources.

### Validation by GWA Studies

#### Validation of Color Index Traits

**Figure 6.**
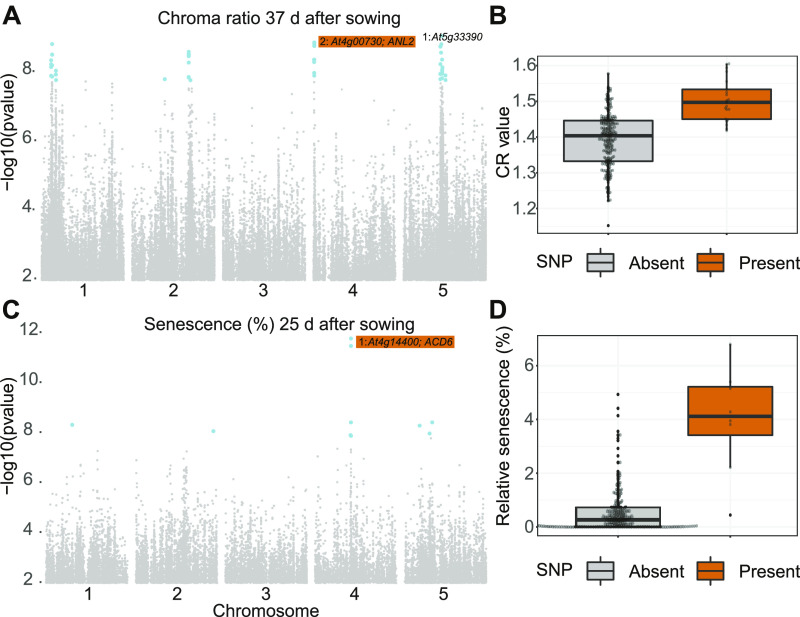
GWA Analyses based on ARADEEPOPSIS Output. **(A)** Results of GWA on the trait “chroma ratio” 37 d after sowing. The log_10_-transformed *p*-value for each SNP is plotted (only SNPs with minor allele frequency >5% are shown). SNPs that are significant after Bonferroni correction are shown in blue. For the two highest-ranking SNPs, the closest gene is indicated. **(B)** Chroma ratio of plants 37 d after sowing, split by accessions carrying the reference and alternative alleles of the SNP close to At2g00730 (*ANTHOCYANINLESS2*, *ANL2*), highlighted in **(A)**. **(C)** Same as **(A)** for the trait “relative senescence” 25 d after sowing. At4g14400 corresponds to *ACCELERATED CELL DEATH6* (*ACD6*, see text). **(D)** Relative senescence 25 d after sowing, split by accessions carrying the reference and alternative alleles of the SNP close to *ACD6*, highlighted in **(C)**.

Often, the ultimate purpose of phenotypic measurements is to relate them to genetic data to understand the underlying genetic determinants. We therefore wanted to test whether ARADEEPOPSIS provided sufficiently high-quality data to identify genetic associations in Arabidopsis by GWA studies, relying on the 1001 genomes variant information (The 1001 Genomes Consortium, 2016). Instead of morphometric traits such as size and growth rate, which are usually highly polygenic and therefore difficult to test, we wondered if we could make use of additional layers of image information provided by ARADEEPOPSIS as a proxy for physiological parameters. We hypothesized that RGB color information of the plant, that is, the respective pixel intensities in the three color channels, would provide information on anthocyanin content and hence on the physiological state of the plant, as it was previously shown that tissue color highly correlates with anthocyanin content (Del Valle et al., 2018). Using the ARADEEPOPSIS segmentations, which accurately reflect the area covered by the rosette, we extracted color information by collecting data from the different RGB channels from the segmented object area, as explained above (Figures 3B and 3C). We then used *LIMIX* (Lippert et al., 2014) to perform GWA analysis on the “chroma ratio” of the rosette area 37 d after sowing. The “chroma ratio” is calculated by dividing the mean intensities from the green channel by half of the sum of the mean intensities from the blue and red channels (Figure 3C). The chroma ratio is therefore inversely correlated with anthocyanin accumulation and thus increases when anthocyanin content is low. We observed strong associations with regions on chromosomes 1, 2, 4, and 5 (Figure 6A). When ranked by -log_10_(p), the second-highest ranking Single Nucleotide Polymorphism (SNP) mapped to chromosome 4, with the closest annotated gene being *ANTHOCYANINLESS2* (*ANL2;* At4g00730). *ANL2* encodes a homeobox transcription factor that has been implicated in controlling anthocyanin accumulation in sub-epidermal tissues (Kubo et al., 1999). Mutants in *ANL2* accumulate less anthocyanins in sub-epidermal leaf tissue. In our data, accessions carrying the alternative allele near *ANL2* displayed a higher chroma ratio, indicative of lower anthocyanin accumulation (Figure 6B). This result demonstrates that ARADEEPOPSIS can noninvasively extract quantitative color index data from whole rosettes that can be applied to genetic association analysis.

#### Validation of Pixel-Level Classifications

Having validated our hypothesis that color profiles of the whole plant area can be used to extract informative traits, we asked if ARADEEPOPSIS would allow the identification of genetic variants significantly associated with the relative amount of senescent or dead tissue during early development. We conducted a GWA analysis, using the relative amount of senescent tissue at 25 d after sowing as the phenotypic trait. This time point is fairly early during the vegetative phase of plant development, and the relative senescent plant area is small compared to later stages (see Figure 2C). We identified several genomic regions that showed significant associations with this phenotype, the most striking of which was located on chromosome 4. The highest-ranking SNP in this region was located within the coding region of *ACCELERATED CELL DEATH 6* (*ACD6;* At4g14400; Figure 6C). *ACD6* has been extensively studied in Arabidopsis and was identified as being associated with vegetative growth, microbial resistance, and necrosis (Atwell et al., 2010; Todesco et al., 2010; Zhu et al., 2018). Our plants were grown on natural, nonsterilized soil from a field site near Zurich, Switzerland (Hu et al., 2018); it is therefore fair to assume that the microbial load and potential biotic stress levels were higher than in standard potting soil. We observed an ∼8-fold difference in median relative senescent tissue per plant (Figure 6D) from ∼0.5% of total segmented pixels in accessions carrying the reference allele to around 4% in those carrying the alternative allele. This result is in line with published studies, which highlighted the role of certain *ACD6* alleles in the early onset of senescence and necrosis in Arabidopsis (Todesco et al., 2010).

### Analysis of Other Plant Species

**Figure 7.**
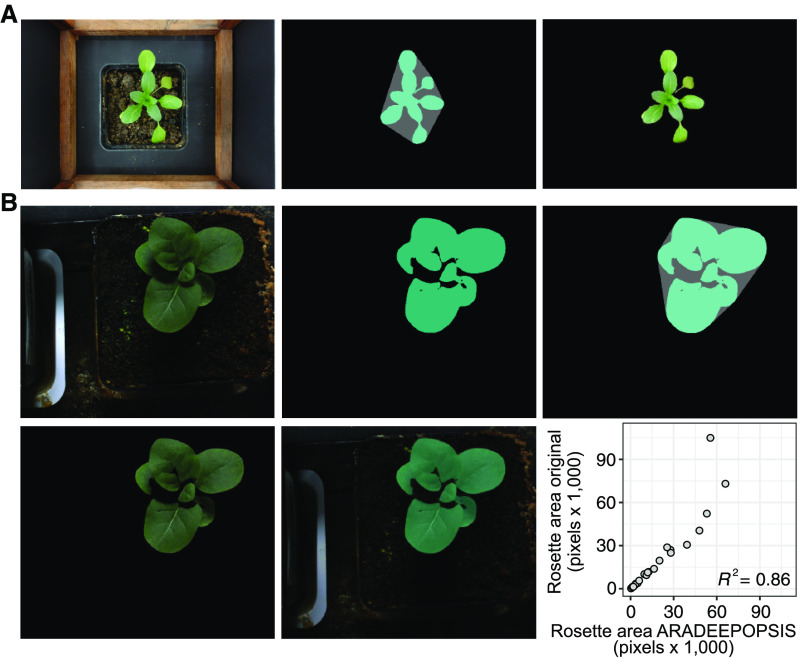
Segmentation of Other Plant Species. **(A)** Segmentation of *Thlaspi arvense*. The left-most panel shows the original image of a *T. arvense* individual. The middle panel shows the segmented mask and convex hull area. The rightmost panel shows the plant accurately cropped from the original image using the mask generated by ARADEEPOPSIS. **(B)** Segmentation of a tobacco (*Nicotiana tabacum*) plant (https://www.plant-phenotyping.org/data sets-download). From top left to center right, panels show the original image, the segmentation mask, the convex hull area, the segmentation, and the overlay of the segmentation and the original image of a representative plant. The scatterplot shows the correlation between ARADEEPOPSIS segmentation and the original ground-truth masks.

While our specific goal was to faithfully segment Arabidopsis rosettes, we wanted to see whether our method would generalize to other plant species with similar overall morphology. We therefore tested ARADEEPOPSIS on top-view images of pennycress (*Thlaspi arvense*), another member of the Brassicaceae, which has a similar leaf shape and phyllotaxis as Arabidopsis but does not form a flat rosette on the ground. Without training a model on images of *T. arvense*, that is, by using the model trained on Arabidopsis, segmentation masks accurately matched the original plant (Figure 7A), showing that ARADEEPOPSIS is robust against variations in plant morphology, as long as these do not deviate from the general architecture that was used in the training set. We also ran the pipeline on images from the IPPN data set (https://www.plant-phenotyping.org/data sets-download; Minervini et al., 2016) on tobacco (*Nicotiana tabacum*), a member of the Solanaceae, which is morphologically quite distinct from Arabidopsis. Segmentations were accurate for most plants and correlated well with the ground-truth masks from the IPPN data set (Figure 7B). We did observe some exceptions for larger plants with substantial shaded leaf areas (Figure 7B; Supplemental Figure 2C).

## Discussion

Here, we present ARADEEPOPSIS, a versatile pipeline to extract phenotypic measurements from small or large sets of plant images in an unsupervised manner. ARADEEPOPSIS can faithfully identify, segment, and measure plants from top-view images of rosettes, such as those of Arabidopsis, using deep learning methodology. Our tool is easy to use, runs on personal computers as well as high-performance computing environments, is robust against variations in overall plant appearance, image quality, and background composition, and generates tabular output, making unsupervised high-throughput image-analysis accessible to a broad community of plant researchers.

### A Fast and Easy-to-Use Segmentation Tool

Our models were trained on sets of 240 (model A) and >1,000 (models B and C) images, respectively, with manually annotated 'ground-truth' segmentations, which required substantial but manageable manual labor. We believe that these investments were warranted, because ARADEEPOPSIS produced accurate segmentations and morphometric measurements that correlated very well with published data; occasional deviations from the originally reported data were in most cases in favor of our pipeline, due to more accurate segmentation by ARADEEPOPSIS. In addition to these pretrained models, ARADEEPOPSIS also allows the use of custom models or sets of presegmented masks for use with the pipeline trait extraction module. Besides being accurate and requiring limited labor, the method is also fast: the fully automated analysis of 100 randomly selected images from our data set took 12 minutes on a laptop computer (24 Gbytes RAM, Intel i5-8250U 1.6 GHz). Depending on available resources such as memory and number of cores, the implementation in *Nextflow* enables straightforward parallelization, allowing for a significant increase in speed when deployed in high-performance computing environments. At the same time, *Nextflow* is agnostic to the computational infrastructure being used, making it straightforward to deploy ARADEEPOPSIS on any type of computer or cloud infrastructure, as long as it fulfills the minimum requirement of 6 Gbytes and 12 Gbytes of memory for single-scale and multi-scale inference, respectively. While training of the model greatly benefitted from the availability of graphics processing units (GPUs), image predictions can also be performed using central processing units in a time-efficient manner.

### Accurate Determination of Plant Health State

ARADEEPOPSIS not only segments rosettes and extracts morphometric parameters, it also allows for the extraction of color channel information, which can be used to make assessments on the anthocyanin content and overall health status of the plant. We have shown that these results can be used, for example, for quantitative genetics approaches (Figure 6).

Besides a simple one-class model (model A) that can segment nonsenescent leaves, ARADEEPOPSIS includes two models that allow segmentation of anthocyanin-rich and/or senescent areas, depending on their color composition. As such, ARADEEPOPSIS is capable of reliably classifying senescent leaves and of distinguishing healthy, green leaves from stressed, anthocyanin-containing leaves. Our models may also be extended to additional classes, depending on the specific needs of researchers and the phenotypes of interest, by including the corresponding training sets.

### Limitations and Shortcomings of the Proposed Models

Many of the images in our training data set had blue plastic meshes as background. This may raise the concern that the model might have learned to classify pixels belonging to the background rather than the plant of interest, that is, to segment the plant as “nonbackground,” which would render the tool unreliable when using it on images with a different background composition. However, by testing ARADEEPOPSIS on images acquired in other phenotyping environments using different backgrounds, and on images of potted plants with substantial algal growth surrounding the Arabidopsis plant of interest, we determined that segmentation of leaves remained accurate and was agnostic to the background (see Figure 5A). Ideally, ARADEEPOPSIS should be used with images that are homogeneous within one set regarding light intensity, exposure time, etc. Segmentation still works for images with various light intensities (Supplemental Figure 4), and plant size and geometry can still be analyzed, but quantification of color intensities based on the original image is no longer comparable in that case.

Our trained models have restrictions with regard to the angle at which the images are recorded and currently perform best for top-view images.

Although we found our models to generalize well to several publicly available data sets (Figure 5), there are also cases where the models misclassify parts of the background (Supplemental Figure 2). Such cases may effectively be avoided by re-training and fine-tuning the model, after adding a small number of annotated images representative of changed image parameters to the training data set. Such training set extensions may either be sourced from manual annotation or by selecting correctly segmented masks produced by the model. We will aim to further diversify the training set for a future iteration of model training and also encourage researchers to use and build upon the training code (https://github.com/phue/models/tree/aradeepopsis_manuscript/research/deeplab) and ground-truth annotations (https://doi.org/10.5281/zenodo.3946393) we deposited for that purpose.

When designing the pipeline, we placed a strong emphasis on the ability for the user to visually assess quality of segmentation at a glance, which we implemented using the *Shiny* framework (RStudio, 2014). This way, users can identify misclassifications early on. Because our pipeline calculates traits independently for each segmentation class, it can extract accurate information for some classes even when others are affected by misclassifications. We demonstrate this in Figure 5C, which shows that ARADEEPOPSIS measurements of class_norm_area correlate well with the area extracted from the IPPN CVPPP2017 annotations (https://www.plant-phenotyping.org/data sets-download; Minervini et al., 2016), despite occasional misclassification of class_senesc, which partially occurred in the A1 image series of the data set (Supplemental Figure 2B).

## Outlook and Perspective

Our study shows that transfer learning is a valuable approach to overcome the phenotyping bottleneck. The model was designed for the analysis of Arabidopsis rosettes but was also able to segment images from other species. We believe that providing a modular pipeline that can be used with different models provides valuable flexibility. For experimental conditions where our models do not perform well, when interest is in other species, or for analysis of other developmental traits than the ones implemented here, researchers can train their own models and simply integrate them into our pipeline to perform trait extraction. These adjustments will require expert annotation of the corresponding images but can be performed by an experienced researcher and trained helpers in a reasonable amount of time. We therefore anticipate that the straightforward usage of ARADEEPOPSIS for trait-extraction with either the provided or custom models combined with direct visualization of the outputs will make semantic segmentation more easily accessible to plant scientists. Moreover, we would like to propose that pre-existing annotations from different research groups and various species be collected into a centralized repository that would serve as a powerful resource for phenomics in plant science and breeding.

ImageNet, the data set that was used to pretrain the baseline model we built upon, contains images of dogs, airplanes, bicycles, etc., but no detailed annotations of plant species. It is therefore remarkable that ImageNet pretrained models already enabled high accuracy for plant segmentation when re-training the last layer with a relatively small set of 240 manually annotated images. Ultimately, the adaptability displayed by ImageNet highlights the potential such publicly available databases and models hold for research and suggests that such resources should be exploited more frequently and more extensively.

## Methods

### Plant Growth and Image Acquisition

Plants (210 genotypes with 6 individuals each; 1260 plants total) were grown in a custom-built growth chamber (YORK Refrigeration), equipped with an environmental control unit (Johnson Controls) and a climate control unit (Incotec), on natural soil (Hu et al., 2018) in long-day conditions (16-h light [21°C], 8-h dark [16°C], 140 µE/m^2^s) with 60% relative humidity and watered twice per week. As light sources, we used R-LMR-5001 light emitting diode panels (Rhenac); the spectrum is shown in Supplemental Figure 5. Seeds were surface-sterilized with chlorine gas for 1 h and sown on September 20, 2018, stratified for 4 d by cooling the chamber to 4°C, and from this point on plants were imaged two times per day. The images were acquired using an RGB camera (IDS uEye UI-548xRE-C; 5 MP) using an automated x-y-z sensor-to-plant system (Hesotech GmbH). Plants were monitored daily for emerging inflorescences, and flowering accessions were removed from the growth chamber to prevent emerging flowers from covering other plants in the top-down images. Plants that had not flowered by December 11, 2018 (12 weeks after release from stratification) were subjected to vernalization for 6 weeks: the chamber was cooled to 4°C (ramped over 8 h). During watering, the temperature was raised to 5°C. This treatment ended on January 21, 2019, after which the plants were exposed to the previous long-day growth conditions. In total 148,792 plant images were acquired and analyzed.

### Training and Validation

We used the *Computer Vision Annotation Tool* (Sekachev et al., 2019) for manual image annotation; custom scripts were used to produce annotation masks from the XML output. The training data are available at https://doi.org/10.5281/zenodo.3946393. The publicly available *DeepLabV3+* (Chen et al., 2017; Chen et al., 2018) code was modified to enable model training on our own annotated training sets. The code used for training, as well as download links for our annotated training data sets, are available here: https://github.com/phue/models/tree/aradeepopsis_manuscript/research/deeplab.

For model evaluation, we split the annotated sets 80:20: 80% of the images were used to train the model and 20% for evaluation.

We used a transfer learning strategy by using a DeepLabV3+ (based on the *xception65* architecture) model checkpoint (Chollet, 2017) that had been pretrained on the *ImageNet* data set (Deng et al., 2009) as a starting point. Training was then implemented in an asynchronous manner using between-graph replication on our in-house *Slurm* cluster, allowing the use of 16 Nvidia Tesla V100 GPUs across 4 compute nodes. We performed model training according to published protocols (Chen et al., 2017; Chen et al., 2018) with the following changes: to account for the number of GPUs, the training batch size was set to 128 and a base learning rate of 0.1 was applied and decayed according to a polynomial function after a burn-in period of 2000 training steps. Images were randomly cropped to 321×321 pixels, and training was stopped after 75,000 iterations. The models were exported to support single- or multiscale inference and are available at https://doi.org/10.5281/zenodo.3946618.

### Implementation

Based on the trained models, we implemented an image analysis pipeline in *Nextflow* (Di Tommaso et al., 2017) that is available at https://github.com/Gregor-Mendel-Institute/aradeepopsis. The workflow is outlined in Figure 4. *Nextflow* allows external pipeline dependencies to be packaged in a *Docker* container, which we provide at https://hub.docker.com/r/beckerlab/aradeepopsis/. The container can be run using *Docker* (Merkel, 2014), *podman* (Containers Organisation, 2020), or *Singularity* (Kurtzer et al., 2017). Alternatively, dependencies can be automatically installed into a *Conda* (Anaconda Inc., 2020) environment.

The pipeline follows a scatter-gather strategy to scatter the input data into equally sized batches of arbitrary size, allowing for parallel processing, after which the results are again collected into a single output table. After the initial splitting, all images in one batch are first converted to TFrecord files using *TensorFlow* (Abadi et al., 2016). The records are then served to the trained *TensorFlow* model of choice in to generate segmentation masks, containing pixelwise classification.

In the next step, we extracted morphometric traits from the segmented images using the *regionprops* function of the Python library *scikit-image* (van der Walt et al., 2014) on a per-class basis. Additionally, color channel information was extracted in the form of mean pixel intensities for each of the channels in the original RGB image, within both the region determined by the segmentation mask and for each class. Based on the color channel means, we calculated chroma indices as follows (Del Valle et al., 2018):

$$Chroma_{base}=\frac{N_{blue}+N_{red}}{N_{green}}$$

$$Chroma_{difference}=\frac{N_{blue}+N_{red}}{2}-\;N_{green}$$

$$Chroma_{ratio}=\frac{N_{green}}{\frac{N_{blue}+N_{red}}{2}}$$

$$BG_{ratio}=\frac{N_{blue}}{N_{green}}$$

$$S_{green}=\frac{N_{green}}{N_{red}+N_{green}+N_{blue}}$$

The results from these measurements were then collected from all processed batches, ultimately resulting in a single table containing 78 traits, listed in the Supplemental Data Set. Based on the segmentation masks in combination with their corresponding input images, the pipeline also produces diagnostic images showing a color-coded mask, the cropped plant region, the convex hull, and an overlay image between the original and the mask.

These single-plant diagnostics are then, in an optional step, merged to produce summary diagnostics using *ImageMagick* (ImageMagick Development Team, 2020), or can be viewed in an interactive *Shiny* (RStudio, 2014) application specifically designed for the purpose of quality control of ARADEEPOPSIS output, allowing for fine-grained inspection of segmentation quality, pixel classification, correlations between traits, as well as time-resolved data visualization if appropriate metadata is provided.

### GWA Studies

We performed GWA analysis on the traits produced by ARADEEPOPSIS using *LIMIX* (Lippert et al., 2014). We used the average of each trait per accession per d and performed GWA analysis by fitting a linear mixed model using *LIMIX*. To associate phenotype and single nucleotide polymorphisms, we used the 1135 genotype SNP matrix and the corresponding kinship matrix, subset to those accessions for which we had trait information. We screened the results for interesting trait-date combinations and followed these using Arabidopsis (*Arabidopsis thaliana*)-specific R tools developed in-house (https://github.com/Gregor-Mendel-Institute/gwaR). The analysis is detailed in the Supplemental File.

### Supplemental Material

**Supplemental Figure 1.** Validation of morphometric measurements against published data.
**Supplemental Figure 2.** Segmentations produced by ARADEEPOPSIS model C.
**Supplemental Figure 3.** Comparison of ARADEEPOPSIS to a dedicated small-scale model.
**Supplemental Figure 4.** ARADEEPOPSIS is tolerant to changes in image parameters.
**Supplemental Figure 5.** Light spectrum applied during the phenotyping experiment.
**Supplemental Data Set.** List of traits resulting from the ARADEEPOPSIS pipeline.
**Supplemental File.** R-scripts used for the analysis of GWAS results.
https://syncandshare.lrz.de/getlink/fiLvKLBUsPKMrj4dLiGckiMa/araDeepopsis_supplement.html

## DIVE Curated Terms

The following phenotypic, genotypic, and functional terms are of significance to the work described in this paper:

ANL2 Gramene: AT4G00730
ANL2 Araport: AT4G00730

ACD6 Gramene: AT4G14400
ACD6 Araport: AT4G14400
