# Erratum to: ARADEEPOPSIS, an Automated Workflow for Top-View Plant Phenomics using Semantic Segmentation of Leaf States

**DOI:** 10.1093/plcell/koab166

**Published:** 2021-07-19

**Authors:** Patrick Hüther, Niklas Schandry, Katharina Jandrasits, Ilja Bezrukov, Claude Becker

Plant Cell **32**: 3674–3688. https://doi.org/10.1105/tpc.20.00318.

In the published version of this article, a superscript number “^1^” should have appeared after Niklas Schandry’s name to indicate that he and Patrick Hüther contributed equally to the work. After Claude Becker’s name, there should have been a superscript number “^2^” to indicate that Drs. Hüther and Becker are the authors for correspondence. The placement of the two superscript numbers was inadvertantly switched during production, so that a “^1^” appeared after Claude Becker’s name and a “^2^” appeared after Niklas Schandry’s name. The article has been updated to accurately reflect the acknowledgment and authors for correspondence. The Publisher apologizes for the error.

